# Vaginal Candidiasis Complications on Pregnant Women

**DOI:** 10.5812/jjm.10078

**Published:** 2014-02-01

**Authors:** Sima Rasti, Mohammad Ali Asadi, Afsaneh Taghriri, Mitra Behrashi, Gholamabbas Mousavie

**Affiliations:** 1 Department of Parasitology and Mycology, Kashan University of Medical Sciences, Kashan, IR Iran; 2 Department of Obstetric and Gynecology, Kashan University of Medical Sciences, Kashan, IR Iran; 3 Department of Statistic, Kashan University of Medical Sciences, Kashan, IR Iran

**Keywords:** Premature Rupture of Fetal Membranes, Low Birth Weight, Vaginal Candidiasis

Dear Editor,

Vaginal candidiasis is one of the most common forms of fungal diseases, that is usually reported in pregnant women which may cause systemic infections in neonate particularly with low birth weight (LBW) and prematurity after delivery (1, 2). *Candida* is an important risk factor of systemic infections in low birth weights infants and the related mortalities (3). Neonatal septicemia produced by *C. dubliniensis *is in the premature infants with LBW has been reported to be successfully treated with amphotericin B (4). There was a tendency towards spontaneous preterm birth reduction among women with asymptomatic candidiasis treated with clotrimazole (5). Screening for eradication of infection during pregnancy may reduce the risk of preterm delivery (2, 6). 

The objectives of this study were to determine the prevalence of *C. albicans *in pregnant women referred to Shabih-Khani Maternity and Gynecology hospital in Kashan, Iran and also to find the effects of infection on the pregnancy including premature rupture of membrane (PROM), preterm delivery and LBW children. This follow–up study was carried out on 150 pregnant women with gestational age of 16-36 weeks with term or preterm delivery referred to Shabih-Khani Maternity and Gynecology hospital of Kashan, from July 2003 to June 2004. 

A questionnaire was provided based on a secure database including demographic data, symptoms of disease, antenatal visits, pregnancy complications and postnatal data. Vaginal secretions were examined by wet mount and cultured on Sabouraud’s dextrose agar (S) (Merck, Germany), Sabouraud’s dextrose agar + chloramphenicol (SC) (Merck, Germany) and teased mount method was used for diagnosis of *Candida *Sp., and then were cultured on Corn meal agar (CMA) (Merck, Germany) and then serum test for differential diagnosis of *C. albicans* was used.

In cases that pseudohyphae and blastoconidia were observed in wet mount and teased mount and more than ten mucoid colonies in culture mediums were grown along with disease symptoms, the pathogen was designated as pathogenic *Candida *sp. and if the chlamydoconidia and germ tubes were detected in CMA and serum tests respectively the infection with *C. albicans *confirmed. The data were analyzed by SPSS version 16 using chi square (continuity correction) and Fischer exact tests. This study was approved by the ethics committee of Kashan University of Medical Sciences.

The prevalence of vaginal candidiasis in pregnant women was 49 (32.7 %). 35% of the patients with preterm labor and 31.8 % with term labor were infected with *C. albicans*. Out of 12 pregnant women with PROM, four women (33.3%) were showed positive results of *C. albicans* infection, while in 138 of the mothers without PROM, *C. albicans* was found in 45 (32.6%) P = 1 (Table 1). Among 29 mothers who had LBW newborns, 5 (17.2%) were positive for *C. albicans* infection, while of 121 mothers with appropriate gestational age newborns, 44 (36.4 %) were showed *C. albicans* infections (P = 0.08). From 40 pregnant women with preterm birth, 14 (35%) was positive for *C. albicans*, but out of 110 with term labor, *Candida* was detected in 35 (31.8%) that statistically was not significant (P = 0.7). 

**Table 1.** Distribution of Pregnant Women According to Candidiasis and Premature Rupture of Membrane

Premature Rupture of Membrane	*C. albicans*		
	Positive, No. (%)	Negative, No. (%)	Total, No (%)
**Present**	4 (33.3)	8 (66.7)	12 (8)
**Absent**	45 (32.6)	93 (67.4)	138 (92)
**Total**	49 (32.7)	101 (67.3)	150 (100)

The results of the study showed that the prevalence of candidiasis among pregnant women in Kashan is lower than that pregnant women in Nigeria (56.3%), but it was higher than those reported in New Guinea 23% (7, 8) and Robat Karim Medical Center, Iran (9). 

No significant relation was found between *C. albicans* infections and preterm labor and PROM and LBW (P = 1, P = 0.08). The results of Hay and Meis is were the same (6, 10). According to the results gained by Kaufman, *Candida* is a risk factor for both systemic infection in low birth weight infants and mortality which was not in consistence with the results of this study (3). 

In spite of high prevalence of candidiasis in the pregnant women, no significant relation was found between candidiasis and preterm delivery and PROM and LBW. Low birth weight of the newborns could be considered as risk factors of *Candida *sp. colonization, so the *Candida* screening of the mothers with preterm labor and proper management helps avoiding the colonization with subsequent risks of invasive Candidiasis.
